# Current observations on shifts in malaria vector biting behavior and changing vulnerability to malaria transmission in contrasting ecosystems in Western Kenya

**DOI:** 10.21203/rs.3.rs-2772202/v1

**Published:** 2023-04-10

**Authors:** Irene Nzioki, Maxwell G. Machani, Shirley A. Onyango, Kevin K. Kabui, Andrew K. Githeko, Eric Ochomo, Guiyun Yan, Yaw A. Afrane

**Affiliations:** Kenyatta University; Kenya Medical Research Institute; Kenyatta University; Kenyatta University; Kenya Medical Research Institute; Kenya Medical Research Institute; University of California; University of Ghana Medical School, University of Ghana

**Keywords:** Malaria vectors, biting behavior, human behavior, Western Kenya

## Abstract

**Background:**

Designing, implementing, and upscaling effective malaria vector control strategies necessitates understanding of when and where transmission occurs. This study assessed the biting patterns of potentially infectious malaria vectors at various hours, locations, and human behavior in different ecological settings in western Kenya.

**Methods:**

Hourly indoor and outdoor catches of human-biting mosquitoes were sampled from 1900 to 0700 hours for four consecutive nights in four houses per village using human landing collection method. The nocturnal biting activities of each *Anopheles* species were expressed as the mean number of mosquitoes landing per person per hour. The human behavior study was conducted via observations and questionnaire surveys. Species within *Anopheles gambiae* and *Anopheles funestus* complexes were differentiated by polymerase chain reaction (PCR) and the presence of *Plasmodium falciparum*circumsporozoite proteins (CSP) determined by enzyme-linked immunosorbent assay (ELISA).

**Results:**

Altogether, a total of 2,037 adult female Anophelines were collected comprising of A*n. funestus s.l*. (76.7%), *An.gambiae s.l*.(22.8%) and *Anopheles* coustani (0.5%). Overall, *Anopheles funestus* was the predominant species collected in Ahero (96.7%) while *An. gambiae s.l* was dominant in Kisian (86.6%) and Kimaeti (100%) collections. PCR results revealed that *An. arabiensis* constituted 80.5% and 79% of the *An.gambiae s.l* samples analysed from Ahero and Kisian respectively. *An. gambiae s.s* (hereafter *An.gambiae*) (98.1%) was the dominant species collected in Kimaeti. All the *An. funestus s.l* samples analysed belonged to *An. funestus s.s* (hereafter *An. funestus*). Indoor biting densities of *Anopheles gambiae* and *An. funestus* exceeded the outdoor biting densities in all sites. The peak biting occurred early morning between 0430–0630 hours in the lowlands for *An. funestus* both indoors and outdoors. In the highlands (Kimaeti), the peak biting of *An.gambiae* occurred between 0100–0200 hours indoors. Over 50% of the study population stayed outdoors from 1800 to 2200 hours and woke up at 0500 hours coinciding with the times highest numbers of vectors were collected. The sporozoite rate was higher in vectors collected outdoors, with *An. funestus* being the main malaria vector in the lowlands and *An. gambiae*in the highland.

**Conclusion:**

The study shows heterogeneity of Anophelines distribution, high outdoor malaria transmission, and peak biting activity by *An. funestus* (early morning) when humans are not protected by bed nets in the lowland sites. Additional vector control efforts targeting the behaviors of these vectors i.e using non-pyrethroids-based indoor residual spraying and spatial repellents outdoors are needed.

## Background

Wide-scale use of implemented vector control tools; long-lasting insecticidal nets (LLINs) and indoor residual spraying (IRS) have led to a substantial decline of malaria burden in sub-Saharan Africa in recent years [1]. However, progress has stalled since 2015 with the majority of African countries including Kenya experiencing persisting malaria transmission even with universal LLIN use and limited IRS deployment [1, 2]. It appears a variety of factors are impeding the expected decrease in the incidences of malaria, for instance, the observed stagnation is hypothesized to be driven by the recent prevalence and strength of pyrethroid resistance that has now been documented in almost all sub-Saharan African countries [3, 4]. Additionally, malaria vectors have shifted their behaviors to reduce exposure to insecticides [5, 6]. Such changes in vector populations threaten progress toward malaria elimination targets [7, 8]. Extensive investigations have been made on vector responses to control tools [3, 9, 10], however, under the current vector control conditions detailed studies are required to understand the prevailing nocturnal human activities and vector biting behavior dynamics.

The indoor interventions rely on vector nocturnal human biting behavior. Historically, the primary malaria vectors *An. gambiae* and *An. funestus* feed entirely indoors with a late-night peak biting activity [11]. This behavior coincides with the time most people are indoors and asleep. However, following the upscale of control tools, the *Anopheles gambiae* has been observed to adjust its behavior to biting before bedtime and outdoors [12–15]. Behavioral avoidance refers to any form of modification in feeding and resting that leads to vectors avoiding/circumventing the lethal doses of insecticides [16]. There is growing evidence of malaria vectors shifting their biting behaviors towards times and places where people are not protected [17, 18]. Host choice and resting patterns have also been observed to change to evade ITNs [18]. For instance, in Kenya, the peak biting time of *Anopheles funestus* has been observed to shift from the classical mid-night feeding time (0100–0200 h) to the early evening and morning hours (0500–0800 h) following the ITNs use and indoor residual spraying [19, 20].

The use of long-lasting insecticidal nets represents a powerful barrier against malaria vectors feeding and resting indoors. However, its efficacy is limited when the human populations are not in bed during the period when vectors are seeking a blood meal [21]. The livelihood activities such as early morning/ night outdoor work for instance farming and security jobs and other cultural practices are potential risks for malaria transmission since exposure to vector bites occurs during these times when unprotected people and vector biting activities overlap in time and space [22]. Thus to achieve elimination, it is critical to understand changes in both the local vector-biting behaviors and where and when persons are exposed to these vectors [23].

Effective mosquito control strategies rely on understanding vector behavior and ecology in combination with the epidemiology of the disease in humans [24]. The majority of studies in Kenya on vector surveillance have focused on vector behavior with less or no attention to human habits and sleeping patterns from different eco-epidemiological settings. Therefore, this study aimed to assess the indoor and outdoor vector biting behavior and human habits and sleeping patterns that may contribute to persistent malaria transmission in Western Kenya. This knowledge is key when evaluating the likely success of the current indoor mosquito control strategies and in designing effective interventions considering the local eco-epidemiological context.

## Methods

### Study sites

This study was carried out in Ahero (0°.11′S, 34°.55′E, altitude 1162 meters above sea level), Kisian (00.0749°S,034.6663° E at an altitude of 1,137–1,330 m asl) and Kimaeti (00.54057°N, 034.56410°E, altitude 1386–1,545 m above sea level). Ahero and Kisian sites are in the lowland plains located adjacent to Lake Victoria in Kisumu County. Ahero is a flood-prone semi-arid area to the south of Kisumu City while Kisian is relatively wet and lies north of Kisumu City. *Plasmodium falciparum* comprises over 90% of malaria infections in Western Kenya. The three malaria vector species, namely *An. arabiensis*, *An.gambiae*, and *An. funestus* are present, with *An. arabiensis* being the dominant species in Ahero (Ototo et al. 2015, Degefa et al. 2017). Kimaeti is located in a malaria epidemic-prone highland area in Bungoma county, western Kenya region. The three malaria vectors are present in the highlands with *An.gambiae* and *An. funestus* being the dominant species depending on the season [28, 29]. The highland and lowland sites of western Kenya have different levels of insecticide resistance (Owuor et al. 2021).

Western Kenya region experiences a bimodal rainfall pattern, with the long rainy season (April—July) which is followed by increased malaria incidence and peak transmission. The short rainy season occurs from October-November. The hot and dry season is from January to March which marks the lowest transmission period [2].

## Mosquito collection

Mosquitoes were collected by the human landing collection (HLC) method in four fixed houses separated by at least 300 m for four consecutive nights. Collections were done three weeks after the long rainy season in July 2021. The HLC was performed by male adult volunteers, who acted as both bait and collector. The volunteers were organized into 3 teams. Each team consisted of 4 individuals, where 2 collected indoors and the other 2 outdoors in each homestead. Before the study, all volunteers were trained in the HLC techniques. Briefly, the volunteers were provided with a flashlight, labels, mouth aspirator, and mosquito-holding cups. The collectors sat on a chair either indoors or outdoors with the lower part of their legs exposed and captured mosquitoes as soon as they landed on them. The mosquitoes were placed in holding cups that had been pre-labeled with the house number, hour of collection, and location (indoors or outdoors). The volunteers collected mosquitoes for 45 min and had a 15 min break every hour. There were two collection shifts: one collector worked from 1800 to 2400 h during each collection night, followed by the second collector from 2400 to 0700 h. A supervisor was assigned to coordinate the collection activities and carry out random spot checks during the collection nights for any challenges and to keep the collectors awake. All volunteers were provided with anti-malaria prophylaxis one week before the start of the study to avoid the risk of contracting malaria during the collection period. Anopheline mosquitoes were sorted morphologically according to the identification keys described by [30] the next morning.

### Anopheline species molecular identification

The legs, wings, and abdomen of morphologically identified *Anopheles gambiae s.l* and *Anopheles funestus s.l* specimens were used for DNA extraction using the ethanol precipitation method following the protocol developed by Collins et al. (1987). The sibling species of *An.gambiae s.l*, and *An. funestus s.l*, complexes were distinguished using conventional polymerase chain reaction (PCR)[32].

## Detection of sporozoite infectivity

The remaining carcasses (head and thorax) of individual mosquitoes were used to detect the presence of *Plasmodium falciparum* sporozoites using the enzyme-linked immunosorbent assays (ELISA) method following the protocol described by Wirtz et al. (1987).

## Human behavior survey

Human activity and sleeping patterns were assessed during the same period when vector collections were carried out. A questionnaire survey was administered to 150 randomly selected household residents living in the study areas. The inhabitant’s social-demographic data was captured in the questionnaire, including the time they went indoors, the activities and cultural practices that kept them out at night, when they retired to bed, when they woke up in the morning and when they left their houses for outdoor activities. In addition, data on bed net ownership and utilization by the households and other intervention tools used for protection from mosquito bites were recorded.

### Data analysis

Human-biting rates (HBRs) for each *Anopheles* species were calculated as the mean number of mosquitoes collected by HLC per person per night (m/p/n) separately for indoor and outdoor venues, i.e. HBR = no. of mosquitoes collected/no. of nights/no. of collectors [34]. The degree of endophagy (proportion of female mosquitoes of a given species that bites indoors) was calculated as indoor HBR1800→0600 h/ (indoor HBR1800→0600 h + outdoor HBR1800→0600 h) while exophagy (proportion of females that bites outdoors) was calculated as outdoor HBR1800→0600 h/(out-door HBR1800→0600 h + indoor HBR1800→0600 h) [35, 36]. The nocturnal biting activities of each Anopheles species was expressed as mean number of each Anopheles species landing per person per hour indoors or outdoors. The number of mosquitoes caught each hour is assumed to represent the number of mosquitoes attempting to feed on humans for the same period. The number of mosquitoes caught biting humans during the different hours of the night, the indoor and outdoor human biting rates of the *Anopheles gambiae s.l* and *An. funestus* group of mosquitoes for the whole sampling period were compared within the sites using a non-parametric test Kruskal-Wallis. The sporozoite infection rate expressed as the proportion of mosquitoes positive for *Plasmodium* sporozoite was calculated by dividing the number of sporozoite positive mosquitoes by the total number of mosquitoes assayed. The analysis was done using open source software, R Version 4.1.3 (2022).

#### Ethical statement

Ethical approval was obtained from the Kenya Medical Research Institute Scientific and Ethics Review Unit (SERU) prior to the start of the study. Household owners, village, and relevant County authorities were sensitized and their permission obtained, while the privacy of the individual participants and household members were highly protected. Volunteers involved in human landing collections were selected from the local community to facilitate acceptance from residents. Informed consent was obtained from each volunteer who were trained to collect landing mosquitoes to minimize the risk of malaria transmission. Participants were screened for malaria parasites and given malaria prophylaxis drugs to protect them from acquiring the disease if they were bitten by infected mosquitoes during the collection. All experiments and methods were carried out in accordance with the relevant guidelines and regulations of SERU.

## Results

### Anopheline mosquito species composition and abundance

Overall, 2,037 *Anopheles* females were collected from the three sites during the study period. Of these, 76.7% (n=1,565) were members of the *An. funestus* group, 22.8% (n=465) belonged to the *An. gambiae s.l* and the remaining 0.5% (n=7) belonged to the An. coustani group (Table 1). *Anopheles funestus* group was most abundant 96.7% (n=1570) followed by An. gambaie *s.l* 3% (n=45) and An. coustani 0.5% (n=7) in Ahero. Out of 351 *Anopheles* females collected in Kisian, 86.6% (n= 304) were *An. gambiae s.l* and 13.4% (n=47) were *An. funestus*. In Kimaeti village all the mosquitoes collected were *Anopheles gambiae s.l*. (n=116). Overall, 58.8% (95% CI: 57–61) of the mosquitoes were captured indoors and 41.2% (95% CI:39–43) outdoors.

Molecular identification confirmed all the *An. funestus s.l* assayed from Ahero and Kisian sites to be *An. funestus*. *An. arabiensis* was most abundant in the lowland sites [Ahero 80.5% (95% CI: 68.4–92.6), Kisian 79% (95% CI: 75.2–85.1)] followed by *An. gambiae* [19.3 % (95% CI:7.4–31.6) and 21% (95% CI: 14.8–24.8) respectively]. In Kimaeti *An. gambiae* [98.1% (95% CI: 95.5–100.7)] was the dominant species followed by *An. arabiensis* [2% (95% CI: 0.7–4.5)]. Ahero had the highest *An. funestus* density (*p* < 0.021) while the *An. gambiae* density was significantly higher in the highland site (Kimaeti) than in any other place (*p* < 0.0001).

### Hourly biting patterns of Anophelines

**Figure 1:**
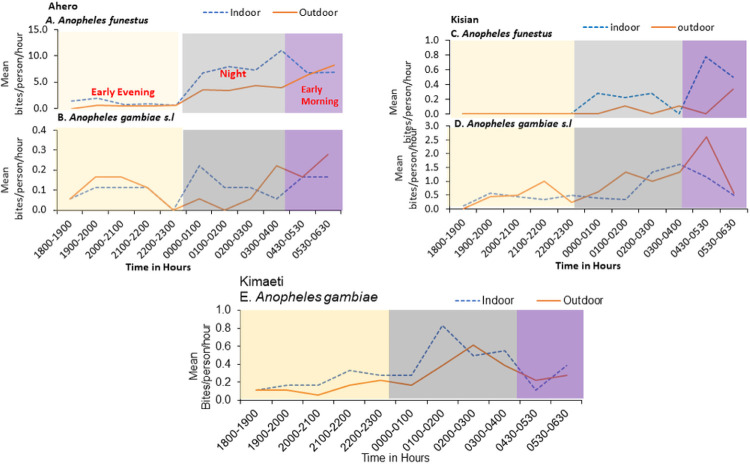
Mean hourly human-biting patterns of the *Anopheles* species in A & B: Ahero (*An. funestus s.l* and *An. gambiae s.l*); C&D: Kisian (*An. funestus* and *An.gambiae s.l*) and D: Kimaeti, (*An. gambaie*).

The man-biting activity of *An. funestus* in Ahero was dawn to dusk both indoors and outdoors with gradual peaks from midnight (0000–100 h) (Mean 7.9 bites/person/hour) and a maximum peak at dawn (0300 to 0400 h) (Mean 11.0 bites/ person/hour) indoors. *An. funestus* showed a steady increase late morning with a peak biting activity at 0530–0630 h (8.2 mean bites/person/hour) outdoor (Figure 1A) when people were out of bed. The biting activity of *Anopheles arabiensis* was generally higher outdoors than indoors with two peaks indoors at midnight and another one late morning 0500 to 0630 h (Mean 0.2 bites/person/hour). The increased outdoor biting activity was observed early evening between 1900–2000 h and this was pronounced in the late morning between 0530–0630 h (0.3 bites/person/hour; Figure 1B).

On the other hand, *Anopheles funestus* in Kisian showed a steady increase in late morning activity with a peak biting activity at 0430–0530 h (Mean, 0.8 bites/person /hour) indoors. The outdoor peak biting activity began at 04:30 to 0630 h (Mean, 0.4 bites/person/hour; Figure 1 C). The biting activity of *An. gambiae s.l* was pronounced at the end of midnight indoors (Mean, 1.6 bites/person/hour, Figure 1D). The outdoor biting activity was bimodal with an early and smaller peak at 2100–22:00 h, and a major peak late morning at 0430–0530 h (Means, 2.6 bites/person/hour; Figure 1 D) which decline progressively during the morning.

**Figure 2:**
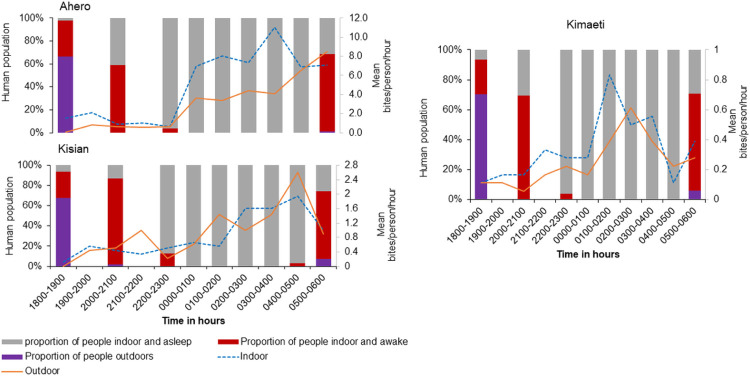
Indoor and outdoor mean hourly biting rates of *Anopheles* mosquitoes with the proportion of people outdoors, indoors and awake, and indoors and asleep throughout the night in A: Ahero, B: Kisian and C: Kimaeti

In the highland (Kimaeti), the biting activity of *An. gambie s,l*.(mostly *An.gambiae s.s*) indoors was bimodal with a major peak at midnight 0100–0200 h (Mean, 0.8 bites/person/hour) (Figure 1 E) when people were asleep and another one early in the morning 0300–0400 h (Mean, 0.6 bites/person/hour) when people were likely to be awake (Figure 2 C). The outdoor activity peaked late in the midnight at 0200–0300 h.

### Anopheline indoor and outdoor biting activity

Overall, the majority of *An. funestus* collected from Ahero and Kisian exhibited endophagic behavior (preference to feeding indoors) (Ahero, 62% and Kisian, 78.7%; Table 2) while *An. gambiae s.l* (mostly *An. arabiensis*) preferred feeding outdoors (exophagy) (Ahero, 51.1% and Kisian, 56.7% respectively; Table 2). Low numbers of *An.coustani* were collected in Ahero (n=7) majority were collected outdoors (5/7). The indoor HBRs of *An. funestus* was higher than outdoor HBR in Ahero (52.3 vs 32.1 mosquitoes/person/night (m/p/n) and Kisian (2.1 vs 0.5 m/p/n respectively). The HBR for *An.arabiensis* was slightly high outdoors than indoors in Kisian (10 vs 7 m/p/n) while in Ahero it was similar for both indoors and outdoors (1.2 m/p/n).

In the highland site (Kimaeti), 57.8% of *An. gambiae s.l*., (mostly *An.gambiae s.s*) collected was higher indoors clearly indicating the preference of this species to feed indoors (endophagy). The indoor HBR for *An.gambiae s.l*., was 3.7 m/p/n and 2.7 m/p/n outdoors.

### Sporozoites infectivity rates

In total, 489 *An. funestus*, 337 *An. gambiae*, 51 *An. arabiensis* and 7 An. coustani samples were tested for the presence of *Plasmodium falciparum* circumsporozoite protein (CSP). Overall, four (4) samples (2 Ahero and 2 Kimaeti) tested positive giving an infection rate of 0.4 (2/474) in Ahero and 1.9 (2/105) in Kimaeiti. In Ahero only *Anopheles funestus* mosquitoes collected indoors (0.3) and outdoors (0.5) were positive for P. falciparum CSP (Table 3). In Kimaeti, CSP was detected in the indoor and outdoor *An. gambiae* collections with infectivity rates of 1.5 and 2.6 respectively. No CSP positives were detected in *An. arabiensis* and An. coustani samples assayed and also for mosquitos collected from Kisian (*n=244*).

### Human exposure to mosquito bites and protection by LLINs

The survey showed that LLIN use was high across the three study sites, 91% in Kisian, 99% in Ahero and Kimaeti with 96.6% of the households having at least one LLIN. Longlasting insecticide nets was the primary prevention method against mosquito bites and malaria infection. Overall, over 50% of the study participants reported to have stayed outdoors or outdoors and indoors until 2100 h. About 77% of the respondents reported going to bed by 2200 h. In Ahero 54% of pre-school-aged children had gone to sleep and 35% of school-going children, 86% of adult women in Ahero, 46% Kimaeti none in Kisian had gone to sleep by 2200hrs while 14% men were asleep by 2200hrs. Overall, at 23:00hrs, majority 93% of the respondents were asleep while 7% were awake indoors.

Across the study sites, it was observed that waking up time was between 0400 hrs and 0700hrs. About 10% of respondents were awake but indoors early morning 0400hrs in Kisian and Ahero coinciding with the time of high mosquito bites (Figure 2A&B). At 0500hrs about 60% were awake and outdoors across the three sites, nearly all exposure to malaria vectors peaked at this time outdoors (Figure 2).

The main activities that kept people outdoors in the evening between 1800 – 2000 hrs included household chores, praying, selling at grocery stores and social gatherings. Night vigils, and watching television after dinner were reported to keep the majority of men awake longer than their female counterparts. Respondents woke up early morning for instance women to prepare breakfast and children to go to school, milking, and doing other domestic chores. Agricultural activities were also the main reason why people woke up early in the morning in particular Ahero where rice plantation is the main activity.

## Discussion

Understanding the biting behavior of malaria vectors and the period, location and frequency at which humans are exposed to infectious mosquito bites in the field plays a crucial role in the fight against malaria. This study outlines the array of *Anopheles* nocturnal biting activity in different eco-epidemiological settings (highland and lowland areas of western Kenya), with data on human behavioral that influence when and where disease transmission may occur. Overall, *Anopheles funestus*, *Anopheles gambiae* s.l and *Anopheles* coustani were found to be the three human-biting *Anopheles* species occurring both indoors and outdoors. *Anopheles funestus* and *An. gambiae* were the dominant vectors biting man indoors while *An. arabiensis* and An. coustani were more likely to bite outdoors. The study further revealed early evening and late morning biting behaviors both indoors (when people are still active and unprotected by LLINs) and outdoors. These behaviors have implications for the risk malaria transmission and the effectiveness of interventions particularly those that target human-feeding vectors indoors.

The study observed *Anopheles funestus* being a predominant vector biting humans in Ahero while *An. gambiae* s.l prevailed in Kisian and Kimaeti. The difference in species abundance could be attributed to the type of breeding habitats available in the study sites, season, degree of predisposition to bite man, and scaling up of insecticide-based intervention [37–40] and mosquito sampling method employed [37,41]. For instance, *Anopheles funestus* is known to breed in permanent habitats with aquatic vegetation cover [38] typical habitats found in Ahero rice irrigation scheme. *Anopheles gambiae* and *Anopheles arabiensis* prefer breeding in small, sunlit temporary water pools [42] type of habitats found in Kisian and Kimaeti areas [43]. Studies on the distribution of Anopheline species in rice fields have documented a succession between *An. arabiensis* and *An. funestus*, (Chandler et al 1976, Githeko et al 1996). The increased abundance of *An. funestus* indicates a significant contribution of this species in the transmission of malaria in this region despite the widespread use of long-lasting insecticidal nets (LLINs).

*An. funestus* and *An. gambiae* exhibited endophagic behavior, with higher proportions seeking a host indoors than outdoors. These findings corroborate earlier reports from western Kenya documenting that the two primary vectors were more likely to seek hosts indoors than outdoors [14,25,26]. In Ahero (lowland site), *An. arabiensis* expectedly preferred to bite outdoors than indoors. In contrast, a higher proportion of *An. arabiensis* was caught biting indoors than outdoors in Kisian (lowland site), demonstrating that mosquito foraging behavior can vary noticeably in relatively small areas. The outdoor biting activity of this species in Ahero could be largely associated with cattle availability in the region, although this was not quantified in this study. Recent studies in Kisian have observed increased levels of insecticide resistance in *An. arabiensis* caught resting indoors compared to the outdoors [44] this could also explain the observed variations in biting activity. Of concern is the fraction of *An. gambiae* and *An. funestus* observed biting outside the classical time (midnight) and whether these behaviors represent resilience or resistance, as this appears to reduce their chance to encounter indoor interventions (IRS and LLINs)[18,45]. The secondary vector An. coustani was observed to prefer foraging outdoors in Ahero (even though the numbers were very low n=7). Although this vector is not given much attention due to its exophagic and zoophilic feeding preferences [37] it has been reported to be susceptible to *Plasmodium falciparum* infections [26,46,47]. The outdoor human-biting activities observed in the current study also imply that it has a potential role in malaria transmission, pointing to the necessity of integrated vector control strategies (IVM) with a combination of non-chemical and chemical methods for more effective vector management i.e biological larval source management and attractive toxic sugar baits (ATSBs).

The biting behaviors of *An.arabiensis* in the lowland sites revealed an early peak in the evening between 1900–2000h outdoors and intense biting activity late in the morning between 0430–0630h (indoor and outdoor), a time when local people are a wake and not protected by LLINs. Our findings are in agreement with previous studies from the same regions [14,37] and elsewhere in Africa [21,48] observed an increase in outdoor biting of this species lasting between 0300 −0500 h in the morning. The increased biting activity outdoors despite the equal chances of the females to bite the human bait in either of the two locations (indoor vs outdoor) may have risen from its preference to host seek outdoors. *Anopheles arabiensis* is known to be flexible in behaviors and in the presence of LLINs indoors and livestock outdoors, man-vector contact is likely to be minimal as the vector seeks an alternative host [26,37,49]. *Anopheles funestus* was responsible for most vectors biting indoors in the lowlands, these observation corroborates with previous findings in the same region [25]. In contrast to early studies on biting behavior, that reported this vector maintaining its classical biting habits (late night) in some regions [9,10], this study observed a shift from classical to late morning biting activity (0530–0630h) (indoors and outdoors) in both lowland sites. A plausible explanation for extended periods of foraging to late in the morning could be a failure to access the preferred host (human) during the feeding hours (late night), forcing the mosquito to wait for the times the host is unprotected. Previous studies in western Kenya have observed pre-biting resting behavior in *An. funestus*, where the vectors were seen resting on the walls before attacking the host [37,50]. Recent studies have observed shifts in the biting behavior of *An. funestus* after universal LLIN coverage and IRS from its historical biting times (late night) to late morning or daytime biting [14,19,20], however, it’s not clear if this behavior is due to plasticity or has a genetic basis. The observed behavior is worrisome as this species (*An. funestus*) is efficient in malaria transmission [26,51,52] and biting during times people are not protected (indoors and outdoors) presents a gap in protection.

In the highlands (Kimaeti), only *An. gambiae* was collected, with previous studies confirming the species to be dominant in the region [29,44]. This vector showed no change in biting activity as the results indicate that the species preferred feeding indoors with a pronounced activity late at night between 0100–0200 h. Historical studies have reported man to be the principal host for this species unlike its sibling species *An. arabiensis* [11]. The persistence to feeding late at night indoors when people are likely to be protected by LLINs partially can be explained by increased resistance levels observed in this species [27,44]. Machani et al. (2022) investigating the host-seeking activity of highly pyrethroid-resistant *An. gambiae*, when a human bait is protected with a treated LLIN, observed that unlike susceptible mosquitoes resistant mosquitoes attempt to bite a host sleeping under a treated bed net. The late-night biting behavior indoors by *An. gambiae*, implies that compliance to LLIN usage could offer protection from infective bites during this period, as the peaks correspond to the times of sleeping. Of concern, is the small peak observed early morning indoors 0300–0400 h when people are waking up and remained unprotected by LLINs as this could have implications on maintaining malaria transmission indoors.

*Anopheles funestus* and An. gambaie were responsible for malaria transmission both indoors and outdoors in the lowland and highland sites respectively, with majority of malaria infections occurring outdoors. The findings are in agreement with previous studies that observed the two vectors to be the main drivers of malaria transmission in the region [25,26,29], however contrary to the present study, the early studies reported high infection rates indoors. It’s worth mentioning that high bed net ownership and usage of > 90% was confirmed in all three sites. The reaction of malaria vectors to indoor-based interventions for instance the excite-repellence effects of pyrethroids used in LLIN [19], may force mosquitoes to shift their biting times explaining the increase in outdoor transmission. This phenomenon can be exacerbated by human behavior in areas where people remain outdoors for long periods of time without protection [54]. In this study, over 50% of the population interviewed stayed outdoors or between outdoors and indoors until 2100 h. The majority of the respondents were asleep by 2300 h (93%), the waking time across the sites was between 0400–0700 h morning with about 10% waking up and staying indoors at 0400 h and about 60% observed outdoors in the morning 0500 h. Human behavior coincides with the vector biting patterns observed in this study. Previous reports indicated that people spend more time outdoors before retiring to bed [25] with a high risk of infectious bites from *An. funestus* outdoors. Agricultural practices (rice farming, milking), domestic chores and other social economic activities (selling at grocery stalls) were the main activities that kept people outdoors. Elsewhere electricity has been shown to influence community outdoor activity and sleeping times as people stay up or out of bed for longer in the evening hours, [54,55], although in this study we did not quantify houses with electricity. This can be confirmed in this study, as men were observed watching television indoors and going to social gatherings (to watch football games) for longer hours in the evening. Therefore, the study findings support previous claims that current control strategies focusing on indoor-based interventions may not be enough to eliminate malaria transmission in most endemic regions [56].

## Conclusions

*Anopheles funestus* and *Anopheles gambiae* were responsible for malaria transmission in the region. The shifting in time of biting from classical biting to late morning biting (indoor and outdoor) of *An. funestus* and the early evening outdoor biting of *Anopheles arabiensis* together with the high outdoor malaria transmission could be due to pressure from the LLINs or humans spending more time unprotected outdoors. These findings have important implications for the epidemiology and strategies for the control of malaria in the study area. Additional control strategies are needed to ongoing interventions to better address the issue of residual transmission and reduce indoor and outdoor biting vectors using a more diverse toolbox with integrated vector management (IVM) strategies.

## Figures and Tables

**Table 1:** Summary of *Anopheles* species collected indoors and outdoors at different times of the night in Ahero, Kisian and Kimaeti villages.

Site	Anopheles species	Indoor				Outdoor				Total
		Early night (18–22h)	Night (00–03h)	Early morning (03–07h)	Total	Early night (18–22h)	Night (00–03h)	Early-morning (03–7h)	Total	
Ahero	*An. funestus s.l*	104	394	443	**941**	41	204	332	**577**	**1518**
	*An.gambiae s.l*	7	8	7	**22**	9	2	12	**23**	**45**
	*An. coustani*	1	0	1	**2**	1	0	4	**5**	**7**
	***overall***	**112**	**402**	**451**	**965**	**51**	**206**	**348**	**605**	**1570**
Kisian	*An. funestus s.l*	0	14	23	37	0	2	8	**10**	**47**
	*An.gambiae s.l*	35	37	59	131	39	53	81	**173**	**304**
	***Overall***	**35**	**51**	**82**	**168**	**39**	**55**	**89**	**183**	**351**
Kimaeti	*An. gambiae*	19	29	19	67	12	21	16	**49**	**116**
	***Total***	**166**	**482**	**552**	**1200**	**102**	**282**	**453**	**837**	**2037**

**Table 2:** Feeding behaviors of *Anopheles* species collected in Ahero, Kisian and Kimaeti villages in Western Kenya.

Site	Anopheles species		Biting activities		
		Indoor (%)	Outdoor (%)	Protected hours (%)	Non- protected hours (%)
**Ahero**	*An. gambiae sl*	48.9	51.1	33.3	66.7
	*An. funestus sl*	62.0	38.0	57.0	43.0
	*An.gambiae s.s*	66.7	33.3	51.5	48.5
	*An. arabiensis*	31.4	68.6	30.0	62.5
	*An. funestus s.s*	60.4	39.6	58.9	41.8
**Kisian**	*An. gambiae sl*	43.1	56.9	47.0	53.0
	*An. funestus sl*	78.7	21.3	38.3	61.7
	*An.gambiae s.s*	50.3	29.6	59.3	40.4
	*An. arabiensis*	86.1	23.8	22.2	69.4
	*An. funestus s.s*	80.0	13.0	66.7	28.6
**Kimaeti**	*An. gambiae sl*	57.8	42.2	33.6	66.4
	*An.gambiae s.s*	62.5	37.5	41.5	58.7

**Table 3:** Indoor and outdoor sporozoite rates of Anopheles mosquitoes collected from Ahero, Kisian and Kimaeti villages in Western Kenya.

Sporozoite infection rate						
Study site	Sibling species	No.	Indoor	No.	Outdoor	Overall (pf+ve)
**Ahero**	*An. gambiae s.s*	**21**	0	**12**	0	**0**
	*An.arabiensis*	**6**	0	**13**	0	**0**
	*An. funestus s.s*	**291**	1 (0.3)	**183**	1(0.5)	**2(0.4)**
	*An. coustani*	**2**	0	**5**	0	**0**
						
**Kisian**	*An. gambiae s.s*	**100**	0	**99**	0	**0**
	*An.arabiensis*	**25**	0	**5**	0	**0**
	*An. funestus s.s*	**12**	0	**3**	0	**0**
**Kimaeti**	*An. gambiae s.s*	**66**	1(1.5)	**39**	1 (2.6)	2(1.9)
	*An.arabiensis*	**2**	0	**0**	0	**0**

## Data Availability

The dataset supporting the conclusions of this article is included within the article

## Abbreviations

HLC: Human landing catches
HBR: Human biting rates
CSP: Circumsporozoite proteins
ITNs: insecticidal treated nets
LLIN: Long-lasting insecticidal nets
IRS: indoor residual spray
